# Does menu calorie labelling cause or exacerbate eating disorders?

**DOI:** 10.1038/s41366-024-01622-3

**Published:** 2024-08-28

**Authors:** Jane Brealey, Rebecca Evans, Amy Finlay, Thomas Gough, Megan Polden, I. Gusti Ngurah Edi Putra, Loukia Tzavella, Rozemarijn Witkam, Eric Robinson

**Affiliations:** Institute of Population Health, University of Liverpool, Liverpool, L69 7ZX UK

**Keywords:** Health policy, Public health

## Abstract

ER and JB were responsible for conceptualisation and study design. JB screened prospective eligible studies, conducted the literature review and wrote the first draft of the manuscript. RE, AF, TG, MP, IP, LT and RW reviewed the literature and contributed to writing. All authors contributed to the manuscript writing, revision, editing, and approved the submitted version.

In April 2022 mandatory calorie labelling in the Out of Home Food Sector (OHFS) was implemented in England as part of the UK government’s strategy to tackle obesity. The policy was designed to provide the public with the information ‘to make healthier decisions’ and requires businesses with 250 or more employees selling food for immediate consumption to display the energy content of unpackaged food and non-alcoholic drinks in kilocalories (kcal), alongside contextual kcal reference information [1].

Previously mandatory calorie labelling on menus has been introduced in the OHFS in parts of the US, Canada and Australia between 2018 and 2019. Calorie labelling is likely to have modest effects on consumer behaviour in terms of kcal purchased [2]. Calorie labelling may also promote product reformulation, with one meta-analysis finding calorie labelling in the OHFS was associated with a statistically significant reduction in kcal (-15kcal) per dish [2].

An Office for Health Improvements and Disparities survey of the general public reported that the inclusion of calories on menus in the OHFS was supported by 79% of the UK population [3]. However, calorie labelling has received some criticism since its implementation. For example, an analysis of Twitter responses to the introduction of calorie labelling found that 71.4% of 276 respondents expressed a negative sentiment towards the policy [4]. This in part reflected public concern about the impact of the policy on people with eating disorders (EDs). Calorie labelling policy has been criticised by BEAT, the UK’s leading ED charity. It has been suggested that the policy increases the vulnerability of those at risk from an ED, exacerbates ED symptoms for those already diagnosed, and perpetuates weight stigma [5].

What does the research say about the potential influence of calorie labelling on people with an ED? Until recently there has been a lack of studies addressing this, but a small number of new studies have attempted to answer this question. Four studies have examined the opinions and experiences of people with EDs using qualitative methods (see Table 1). Recurrent themes describe how calories on menus: can lead to a hyper-fixation on calories, restrict food freedom (meals are chosen for their calorific value rather than what was actually wanted or appropriate for hunger levels), reduce eating out opportunities, increase feelings of anxiety, guilt and shame around food choices, and inhibit ED recovery. Some participants in these studies expressed anger over the messaging from ‘trusted’ public health authorities on the normalisation of calorie counting, because calorie counting had played a pivotal role in the development of their ED [6, 7]. Although these qualitative studies predominantly list negative impacts on those with EDs, some positive themes were also identified: calorie labelling can increase feelings of reassurance, control and accountability of food eaten [7, 8], and for some, the information provided may help to reduce overconsumption and the subsequent guilt [9]. Although for others, this element of control was seen as negative, as it reinforced disordered eating behaviours [7].

**Table 1:** Studies reporting qualitative and quantitative data relating to participants experiences and support vs. opposition for calorie labelling.

Study	Sampling approach	Participant information^a^	Methodology	Results relating to eating disorders
Qualitative studies				
Duffy et al. [6]	11 participants Online interview England Must have experienced an ED in past or currently have an ED (self-reported)	5 recovered from ED (100% Female) 6 current ED (83% Female) Age 18+	Open-ended questions examining participants experiences and opinions of calorie labelling ‘Can you talk me through a recent experience you have had of calories on menus and the impact (if any) this had on your eating disorder symptoms?’ ‘How has the introduction of the policy impacted your eating disorder symptoms?’ ‘Have you found any strategies (if required) that have helped at this time?’ Self-report	Negative effects of calorie labelling Six themes established, all themes found negative impacts of calorie labelling for those with EDs: Theme 1: A personal attack on those with EDs Theme 2: Placing calories in the spotlight Theme 3: Normalising calorie counting Theme 4: Making appropriate meal choices more burdensome Theme 5: Negatively impacting ED symptomatology through loudening ED thoughts and altering food choices Theme 6: Strategies now needed to deal with calorie labelling Benefits of calorie labelling No benefits were identified
Frances et al. [7]	399 participants Online survey UK Must have experienced an ED in past or currently have an ED (self-reported)	91% Female, 5% Non-binary, 2% Non-conforming, 2% Male, <1% preferred not to say Age 16+	Open-ended survey questions examining experiences, challenges and impacts of calorie labelling Self-report	Negative effects of calorie labelling Five out of the six themes established found negative impacts of calorie labelling for those with EDs: Theme 1: Negative impact on relationships /increased tension when eating out Theme 2: Exclusion and increased isolation from eating out, increased shame Theme 3: Restricted food freedom Theme 4: Dis/embodiment; food chosen was not what was actually wanted/needed, but selected because of the calories Theme 5: Anger that calorie labelling is seen as beneficial for health which does not reflect participant experience Benefits of calorie labelling One of the six themes established found positive impacts of calorie labelling for those with EDs: Theme 6: Increased feeling of control, responsibility and accountability for their recovery / helping reduce anxiety
Putra et al. [8]	1273 participants Mixed methods Online survey UK, fluent English Must have past or current GP diagnosis of mental health condition (including ED)	583 with an ED (75% Women, 21% Men, 4% Non-binary/ other) Age 18+ Mean age 32	Free-text responses relating to perceived effects of calorie labelling policy on current ED symptoms Self-report	Negative effects of calorie labelling Two of the four themes established found negative impacts of calorie labelling for those with EDs: Theme 1. Hyper-fixation on calories and potential relapse from ED recovery Theme 2: Negative effects on mood through increased guilt and anxiety, and reducing enjoyment of eating out Benefits of calorie labelling Two of the four themes established found positive impacts of calorie labelling for those with EDs: Theme 3: Increased feelings of reassurance and feeling informed about foods Theme 4: Feeling in control of eating and enabling planning of food intake
Raffoul et al. [9]	13 university students Campus-based menu labelling study Semi-structured one-to-one interviews Canada ED status not reported. Please insert new subheading below this row titled 'Quantitative studies'	10 Women, 3 Men Mean age 19	Closed and open-ended questions examining participants experiences and feelings about calorie labelling ‘Can you name a specific time when you saw calorie labels and tell me how you felt or reacted?’ ‘Can you tell me about how seeing labels with calorie content makes you feel, considering your relationship with food?’ Self-report	Negative effects of calorie labelling Two of the four main themes established found negative impacts of calorie labelling: Theme 3: Labels affect their own or others’ relationship with food by exacerbating EDs, causing shame around eating and leading to calorie fixation Theme 4: Labels lead to an increase in disordered eating thoughts and feelings of shame and regret around food choices Benefits of calorie labelling Within Theme 4 some participants also described how labels may help reduce shame around food choices by providing information and thus reducing ‘overconsumption’ and the associated guilt
Quantitative studies				
Frances et al. [7]	399 participants Online survey UK Must have experienced an ED in past or currently have an ED (self-reported)	91% Female, 5% Non-binary, 2% Non-conforming, 2% Male, <1% preferred not to say Age 16+	One closed question exploring challenges of calories on menus: ‘Have you experienced challenges due to having calories on menus?’ Self-report	91% had experienced challenges because of calories on menus
Putra et al. [8]	1273 participants Mixed methods Online survey UK, fluent English Must have past or current GP diagnosis of mental health condition (including ED)	583 with an ED (75% Women, 21% Men, 4% Non-binary/ other) Age 18+ Mean age 32	Examined acceptability and perceptions of calorie labelling policy through Likert scale questions ‘Businesses like restaurants, fast food outlets and coffee shops should be required to display the calorie content of their foods on menus or menu boards.’ ‘Seeing calorie information on menus or menu boards will make my eating disorder symptoms…’ ‘I will feel anxious if I see calorie information on menus and menu boards when eating out.’ ‘Compared to eating out without calorie labelling information, calorie labelling will make me feel more guilty when eating out.’ ‘Compared to eating out without calorie labelling, calorie labelling will make me feel more afraid about eating out.’ Self-report	43% of participants with an ED agreed or strongly agreed with implementation of calorie labelling policy, 11% were neutral, 46% disagreed or strongly disagreed 55% of participants reported calorie labelling may worsen ED symptoms 63% of participants with an ED agreed or strongly agreed that seeing calorie information would make them feel anxious when eating out 70% of participants with an ED agreed or strongly agreed that seeing calorie information would make them feel more guilt when eating out 52% of participants with an ED agreed or strongly agreed that calorie labelling would make them feel more afraid about eating out
Raffoul et al. [9]	13 university students Campus-based menu labelling study Semi-structured one-to-one interviews Canada ED status not reported	10 Women, 3 Men Mean age 18	Closed and open-ended questions examining participants experiences and feelings about calorie labelling ‘Can you name a specific time when you saw calorie labels and tell me how you felt or reacted?’ ‘Can you tell me about how seeing labels with calorie content makes you feel, considering your relationship with food?’ Self-report	100% identified at least one negative impact of calorie labels on either their own and/or others’ relationship with food 38% stated calorie labels may be harmful for those with EDs or lead to more people developing an ED 85% of participants supported calorie labels and their implementation

^a^Gender/sex recorded as reported in each study.

In these recent studies, quantitative data suggest some support towards the policy; with the one study that measured policy support finding 43% of participants with diagnosed eating disorders agreed with policy implementation (Table 1). When specifically asked about ED symptomatology, 55% (of 583 participants with EDs) reported that calorie labelling may worsen their ED symptoms [8] and 91% (of 399 participants) stated that they had experienced challenges because of calories on menus [7].

But what about measured objective changes to ED symptoms as a result of calorie labelling? To date, there is very limited evidence, however one study attempted to examine negative outcomes from calorie labelling in 2015. In a sample of 299 undergraduate females, considered as at high risk from eating pathologies, Lillico et al. [10] measured eating disturbance scores, affective reactions (anxiety and body image satisfaction), unhealthy weight-related behaviours (binging, restricting calories, exercising excessively) and calorie consumption, before and after calorie labelling was introduced in a university cafeteria. They found no significant differences across all measures when calorie labels were absent or present, and concluded that there were no adverse effects of calorie labels on those at risk of EDs. However, it must be noted that although these participants were considered at high risk of eating pathologies due to their age and sex, they were stratified by Eating Attitudes Test scores rather than a formal ED diagnosis. Further research on the impact of calories on menus is therefore needed to see if these findings translate to those with diagnosed EDs. Likewise, there is now a need for more studies to develop the very minimal evidence base outlined in Table 1, including further research on impacts among those living with obesity and an ED, and understanding impacts among those with different types of ED (including binge eating disorder, anorexia nervosa and bulimia nervosa (see Fig. 1).

**Fig. 1:**
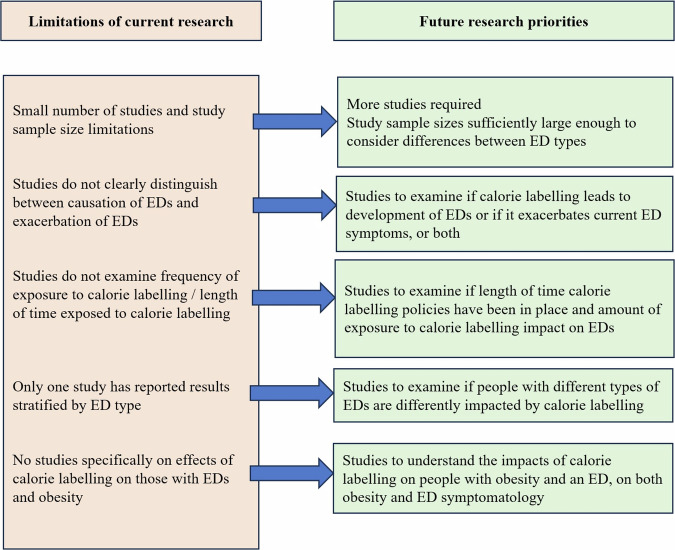
Key research priorities on the impact of calorie labelling on EDs.

The UK government calorie labelling guidance does acknowledge that those with EDs may find seeing calorie information on menus challenging and permits that businesses provide a menu without calories on request, ‘at the business’s discretion’ [1]. However, anecdotal experiences of those with EDs suggests that menus without calories are not always available [11], with a recent observational study finding just 12% of 90 food outlets had a calorie-free menu available on request [12]. Furthermore, the very act of requesting may be distressing for those with an ED by drawing further attention to their ED [11]. Perhaps the best solution would be for all establishments to mandatorily provide menus with and without calories, either online or via QR code [7] and thus eliminate the need to publicly request a menu without calories.

EDs are complex mental health conditions. Many people with EDs also have obesity, and having obesity is also a risk factor for developing an ED [5]; thus large numbers of people that are the ‘target’ of calorie labelling are at risk of harm. Yet despite the perceived negative impacts of calories on menus, many people living with an ED understand the need to address population-level obesity and a significant proportion support calorie labelling. However, this should not be at the detriment of their own health, and should not increase weight stigma or encourage restrictive eating. As with every public health policy, we should aim to ‘first do no harm’. Based on studies conducted to date, we propose it should be mandatory for businesses to provide calorie free menus alongside menus with calories.
